# Numerical and experimental assessment of hydrogen enrichment effects on ci engine characteristics fuelled with dual biodiesel blends: A comprehensive study

**DOI:** 10.1371/journal.pone.0351152

**Published:** 2026-06-05

**Authors:** B. Vishnu Vardhana Naidu, B. Musthafa, M. Raja, S. Sivalakshmi, S. A. Srinivasan, B. Saravanan, Yohanis Dabesa Jelila

**Affiliations:** 1 Department of Mechanical Engineering, School of Engineering, Mohan Babu University, Tirupati, India; 2 Deparment of Automobile Engineering, BS Abdur Rahman Crescent Institute of Science and Technology, Chennai, India; 3 Department of Mechanical Engineering, Government College of Engineering, Salem, India; 4 Department of Mechanical Engineering, Shiv Nadar University, Kalavakkam, India; 5 School of Mechanical Engineering, Vellore Institute of Technology, Vellore, India; 6 Faculty of Mechanical Engineering, Jimma Institute of Technology, Jimma University, Jimma, Ethiopia; GH Raisoni College of Engineering and Management Pune, INDIA

## Abstract

The growing need for energy around the world is putting pressure on established power sources like fossil fuels, making renewable energy solutions more concern. The high calorific value of hydrogen has made it an attractive technique for increasing combustion rates. To get the best possible results from a dual biodiesel blend of juliflora and kapok, this research seeks to improve the hydrogen enrichment ratio. When compared to diesel, the combined effects of 12H_2_ + JK B20 increased BTE by 11.5%, CP by 6.9%, and HRR by 5.9%. The use of H_2_ and JK B20 improved combustion, leading to a decrease of 8.5% in BSFC, 10.9% in CO and HC emissions, 11.5% in smoke, and 14.6% in both. The increase in NOx emissions is a result of the trade-off. The RSM results demonstrate that the test blend B20, which included 12% H_2_ at 100% load, exhibited an attractiveness index of 0.995. An effective and trustworthy prediction framework for optimizing CI engine properties, the ANN model was fitted using experimental data and validated. Sustainable transportation and environmental protection stand to benefit greatly from the synergistic effects of H_2_ enrichment and dual biodiesel.

## 1. Introduction

The world’s economy has relied on fossil fuels for more than a century as its principal energy source. Nevertheless, the hunt for sustainable, renewable, and low-carbon energy alternatives has become imperative due to increasing energy demands, decreasing fossil fuel supplies, and environmental worries linked to greenhouse gas emissions [1]. An attractive alternative that has recently gained traction in this area is biodiesel, which is often produced by trans-esterifying vegetable or animal fats or used cooking oils. It may be used directly in diesel engines without changing the engine architecture because it exhibits fuel qualities like normal diesel. Among biodiesel’s many benefits are its increased cetane number, biodegradability, and lubricity [2]. Palm seeds, Jatropha, and pongamia are the most well-known feedstocks. The juliflora seed has great potential as a source of biodiesel, but it has not been utilized thus far. In terms of biodiesel manufacturing, it provides a number of benefits. The fact that it is less expensive to harvest for farmers than other biodiesel sources is a major plus. Because of its rapid expansion and resistance to high and low pressure, it is a promising material for use in renewable energy and biodiesel [3]. When it comes to CI engines, a handful of studies have focused on the essential qualities of juliflora biodiesel [4]. Debella et al. [5] looked at how well Juliflora biodiesel (B20) mixed with diethyl ether performed and what kind of emissions it produced. The B20DEE10 gets 31.4% BTE, which is almost diesel-like. Low levels of NOx are offset by lower levels of CO2, HC, and CO emissions. Its smoke opacity value is lowest when it is fully loaded. By studying the effects of different DEE concentrations on combustion parameters, the author hopes to find the sweet spot for burning Juliflora biodiesel. The findings show that peak CP increases by 6.5% and HRR increases by 4.7% when using a 10% DEE blend [6]. Results from both computational and experimental studies showed that juliflora B20’s lower blend ratio performed similarly to diesel fuel. Musthafa [7] decided that B20 was the best blend out of the ones they evaluated because it had higher performance emissions characteristics and may be used as a substitute for diesel fuel.

A promising new fuel source, kapok biodiesel has recently emerged from the extraction of kapok oil from kapok seeds. The primary reasons for adopting kapok seeds as a feedstock were their high oil content and their abundant availability [8]. The high bio oil content of kapok seeds made it one of the edible resources that could be used to make high-quality biodiesel [9]. Kapok seeds contain 25–28% oil by weight per fruit and produce an average of 1,285.9 kg/ha per year [10]. Nandhakumar et al. [11] have looked at how a CI engine that ran on kapok biodiesel fared when different nozzle hole geometries were used. The engine specs for the B50 blend have been looked for 50% diesel and 50% kapok biodiesel, and the number of nozzle holes from three to five have increased. Since better fuel atomization and air/fuel mixing were anticipated, this led to 10% higher CP and 32.5 higher HRR in the combustion process. Modifications to engine designs are necessary for running greater biodiesel blends. Pooja et al. [12] tested a VCR engine with 20% kapok biodiesel, they found that compared to diesel, the emissions of HC were 8.4% lower, CO2 emissions were 13.7% lower, and CO emissions were 5.08% lower. Based on the results of the performance investigation, kapok blends have the potential to be a viable alternative fuel for CI engines. Udhayakumar et al. [13] found that CI engines benefit from kapok biodiesel blend B10, which increases BTE by 5% and decreases BSFC at rated load conditions. Kapok biodiesel could be utilized successfully in existing CI engines without any modifications, according to their results. Juliflora biodiesel is rich in oleic acid (monounsaturated fatty acid), which enhances cetane number, improves ignition quality, and provides better oxidative stability, resulting in smoother and more controlled combustion. In contrast, Kapok biodiesel contains a relatively higher proportion of linoleic acid (polyunsaturated fatty acid), which contributes to improved volatility and inherent oxygen content, thereby promoting better air–fuel mixing and more complete combustion. This complementary fatty acid synergy enhances atomization, combustion efficiency, and emission characteristics compared to individual biodiesels.

Blends of conventional diesel with two separate biodiesels, often made from different feedstocks like Jatropha and Kapok, are combined to create a fuel blend called dual biodiesel. This allows for the utilization of synergistic physicochemical and engine performance benefits. Using a CRDI engine in dual fuel mode, Jaiganesh et al. [14] investigated a biodiesel blend of juliflora and waste plastic oil, and they found that the B15 blend improved BTE. Better outcomes of reduced NOx and other emission trade-offs are achieved when B20 blends with EGR. In conclusion, diesel isn’t as good as dual biodiesel with 10% EGR when it comes to performance and emissions. Using juliflora biodiesel blends and a H_2_ additive, Palani and Subramanian [15] investigated how the performance and emissions of dual-fuel diesel engines were affected by the number of nozzle holes. With a 4-hole nozzle, researchers were able to decrease BSFC by 4.28% and enhance BTE by 28.5%. Improved atomization and reduced flame intensity were the outcomes of combining PJB20 with 6 lpm of H_2_. The results indicate that H_2_, which improves combustion strength and atomization, can be added to biodiesel to reduce its drawbacks. The experimental and numerical analysis of the CI engine employing Juliflora-Gulmohar dual biodiesel was carried out by Musthafa and Prabhahar [16]. With a BTE of 25.6%, the JGB20 blend has a BSFC of 0.282 kg/kW.h when operating at full load. Additionally, when comparing JGB20 mix to diesel, the HC, CO, and smoke emission metrics were 4.8%, 16%, and 10% lower, respectively. Srinivasan et al. [17] investigated the features and benefits of developing dual blends of diesel and Juliflora biodiesel, which contain low carbon alcohols. Reduced CO and HC emissions by 34% and 39% compared to diesel, while increasing BTE by 3.8% and improving BSFC by 0.3 kg/kW.h.

In comparison to other gaseous fuels, H_2_ has the fastest flame speed and the widest range of combustibility. Because it has a lower ignition energy, is harmless, and typically improves combustion through faster flame speed. In order to improve engine performance and control emissions, Vijay Kumar [18] studied biodiesel additives that use H_2_ enrichment (HE) and condition monitoring techniques. He discovered that HE enhances biodiesel combustion by increasing BTE and decreasing CO and HC emissions, but it may increase NOx levels because combustion temperatures are higher. Basha et al. [19] examined at how low-carbon fuels’ combustion and flame properties were affected by HE. An earlier start of combustion and a greater peak HRR were detected, indicating that the combustion and flame growth process were aided by H_2_’s stronger physical diffusivity and chemical reactivity. Using HE waste cooking biodiesel, Ahmed et al. [20] improved the CRDI engine’s performance by 7% while reducing BSFC by 11%. One possible fossil fuel alternative that improves engine performance is biodiesel with additional antioxidants and H_2_. Katre et al. [21] examined the effects of HE on a dual-fuel engine running on Karanja biodiesel and a biodiesel mix containing 20% HE. They found that this blend improved combustion characteristics, which in turn increased CP and HRR while reducing HC and CO emissions by 23% and 16%, respectively. Many other researchers also found similar results. Furthermore, Thiru Selvam et al. [22] found that, with the exception of increased NOx levels, engine performance and emissions were greatly improved when biodiesel and HE were used together. The optimal mixture for efficiency and emissions, according to their optimization results, is a mixture of 20.17 percent cottonseed oil biodiesel, 26.99 g/h H_2_, and 5.37% EGR [23]. This study supports the United Nations Sustainable Development Goals, particularly SDG 7 (Affordable and Clean Energy) and SDG 13 (Climate Action), by promoting renewable fuel utilization and reducing engine emissions [24].

### 1.1. Novelty and motivation of the study

A unique contribution of this work is the demonstration that blending Juliflora with kapok biodiesel in dual fuel mode results in enhanced physicochemical properties of viscosity balance and calorific value that surpassing the performance of individual biodiesels. The study introduces hydrogen enrichment as a novel approach to compensate for the relatively lower calorific value and slower combustion of the dual biodiesel blend, resulting in improved thermal efficiency and reduced ignition delay. In order to maximize the synergistic effects of H_2_ and dual biodiesel, which include increased thermal efficiency, faster flame speeds, and longer lean-burn limitations, it is critical to optimize the HER. This optimization strikes a compromise between efficiency, power output, and conforming to strict emissions standards. Thus, for safe, effective, and long-term engine running, the optimal ratio is system-specific and crucial. Unlike previous studies, this work integrates dual biodiesel (Juliflora–Kapok) with hydrogen enrichment and applies both RSM and ANN for simultaneous optimization and prediction, providing a comprehensive evaluation of combustion, performance, and emission characteristics.

## 2. Material and methodology

**Fig 1:**
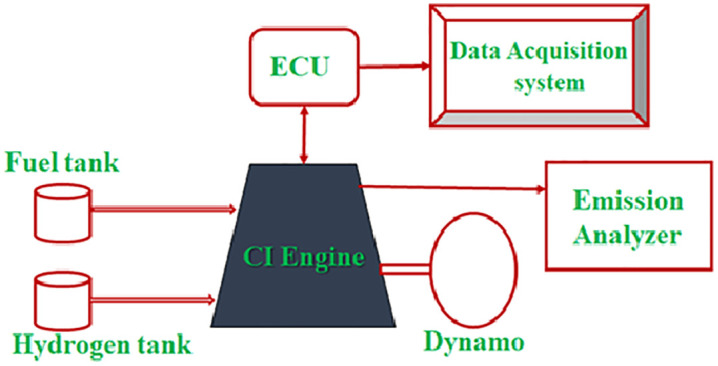
Schematic diagram of test engine.

In this study, transesterification is used to produce Juliflora and Kapok biodiesel. JK dual biodiesel is manufactured by mixing it at a volume ratio of 50% by weight and 50% by volume. Next, combine 80% JK B20 with 20% JK dual biodiesel by volume. Biodiesel and its mixtures’ physiochemical properties are displayed in Table 1. Table 2 presents details of the instruments used to test the 4S single-cylinder Kirloskar CI engine’s performance, combustion, and emissions; Fig 1 shows the corresponding schematic. A 17.5:1 compression ratio, 5.2 kW of rated power, and a rated speed of 1500 rpm were its specifications. The engine was operated at a constant injection timing of 23° bTDC and an injection pressure of approximately 200 bar as per manufacturer specifications. The air–fuel ratio varied with load and hydrogen induction. A nozzle supplied H_2_ gas to the inlet manifold on a continuous basis; it was then naturally mixed with air and sent to the combustion chamber. The air–fuel ratio varied with load and hydrogen induction. A nozzle supplied H₂ gas to the inlet manifold on a continuous basis; it was then naturally mixed with air and sent to the combustion chamber. The hydrogen flow rate was measured and regulated using a calibrated gas flow meter (rotameter), ensuring precise control of flow rates at 8, 10, and 12 litres per minute (lpm). A control valve was used to maintain steady and continuous hydrogen supply throughout the experiments, which promoted uniform mixing in the intake manifold under steady operating conditions. The selected hydrogen flow rates (8–12 lpm) were chosen to ensure stable engine operation and avoid abnormal combustion conditions.

**Table 1:** Physiochemical properties of the test fuel.

Fuel properties	Diesel	*Juliflora*	Kapok	JK dual biodiesel	
	B0	B100	B100	B100	B20
Kinematic viscosity (mm^2^/s) at 40^o^C	2.81	6.72	5.30	6.01	3.45
Density (kg/m^3^) at 15^o^C	845	875	872	873.5	850.7
Lower heating Value (MJ/kg)	43.6	39.2	40.5	39.85	42.85
Cetane Number	50	52	60	56	51.2

**Table 2:** Specifications of equipment.

Instrument	Measurement	Range	Accuracy
Load cell	Load (kg)	0–16.4	±0.1
Speed sensor	Speed (rpm)	0-9999	±1
Encoder	Crank angle (°CA)	1- 360°	±1°
Pressure sensor	CP (bar)	0 −250	±0.2
Thermocouple	Temperature (°C)	0-1000	±1
AVL Smoke meter	Opacity (%)	0 - 100	±0.1
AVL Di gas analyzer	CO (% vol) HC (% vol) NO_x_ (ppm)	0–15 0–30000 0 - 5000	±0.01 ±1 ±1

The flow meter was periodically calibrated, and its measurement uncertainty was considered within the overall experimental uncertainty analysis. A control valve was used to maintain steady and continuous hydrogen supply throughout the experiments. To guarantee the study’s dependability, repeatability tests and error analysis were conducted [25]. The accuracy of the test devices, including the gas analyzer, smoke meter, and test engine configuration, was quantified. Through the use of the square root method, the total uncertainty was evaluated.


$$\begin{array}{c} \text{Overall uncertainty }=\;\surd \;{\left[(\text{0}.\text{1}\right)}^{\text{2}}+({\text{1})\text{2}}^{\text{2}}\;+({\text{1})}^{\text{2}}+\;{\left(\text{0}.\text{2}\right)}^{\text{2}}+{\left(\text{1}\right)}^{\text{2}}+\;{\left(\text{0}.\text{01}\right)}^{\text{2}}+\;{\left(\text{1}\right)}^{\text{2}}+\;{\left(\text{1}\right)}^{\text{2}}+{\left(\text{0}.\text{1}\right)}^{\text{2}}]\;=\;\text{2}.\text{25 }\%. \end{array}$$

The combination of repeated trials and uncertainty analysis confirms that the experimental results are reproducible and statistically reliable. The calculated overall uncertainty of ±2.25% applies to all experimentally measured and derived parameters, including brake thermal efficiency (BTE), brake specific fuel consumption (BSFC), cylinder pressure (CP), and emission characteristics (CO, HC, NOx, and smoke opacity). Since these parameters are computed from measured quantities, their uncertainties are inherently accounted for through the propagation of instrument errors. Due to the use of 3D response surface plots, error bars could not be incorporated; however, repeatability tests were conducted and validated using standard deviation analysis.

### 2.1. RSM and ANN modelling

To achieve the defined experimental outcomes, Design Expert-13 software with Box-Behnken Design (BBD) was employed systematically to develop the design of experiments (DOE). The response surface methodology (RSM) framework is used to establish optimal operating settings by assessing load (L) and biodiesel with H_2_ concentration. Before RSM numerical modeling, the experimental dataset is created. Optimization finds the best independent variables by adjusting responsive variables to standards. The experiments provide data on the studied parameters. We developed the approach and findings after identifying the dependent and independent variables. The proposed experiments have used for specifying parameters and to maintain all other settings. The BBD studied load and biodiesel H_2_ concentration 20 times. RSM with quadratic model analyzes Equation 1 inputs. To test model performance, compared engine parameters predicted by model to actual data. Load and biodiesel H_2_ concentration determined engine performance, combustion, and emissions. This research uses surface plots, ANOVA, and R^2^. Based on engine load and biodiesel blend, this RSM analysis revealed optimal operating parameters. Table 3 demonstrates response variable levels and factors.

**Table 3:** Input and level factors.

Variables	Unit	Level		
		−1	0	1
Hydrogen Concentration	lpm	8	10	12
Load	%	0	50	100


$$\begin{array}{c} Y=m_{\text{0}}+\sum_{i=\text{1}}^{n}m_{i}P_{i}+\sum_{i=\text{1}}^{n}a_{ii}{P_{i}}^{\text{2}}+\sum_{i=\text{1}}^{n}\sum_{J=\text{1}}^{n}m_{ij}P_{i}P_{j} \end{array}$$ (1)

**Fig 2:**
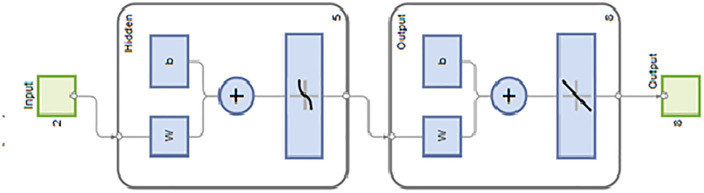
Network Topology layer.

An Artificial Neural Network (ANN) was built using two independent factors and seven dependent variables in this study. The ANN model is built sequentially in MATLAB. This technique involves setting variables, creating neuron layers, training neurons, and testing the learnt model on different topologies. The topology layer of the network, as depicted in Fig 2. Training for each topology continued until the mean squared error dropped below a threshold. To evaluate artificial neural network error, R^2^, mean squared error, and average relative error % are calculated during training [26]. A feedforward backpropagation ANN with two input neurons, three hidden layers, and seven output neurons was developed. The hidden layers used tansig activation functions, and the output layer used a purelin function. The network was trained using the Levenberg–Marquardt (trainlm) algorithm. The ANN hyperparameters, including the number of hidden layers, neurons, and activation functions, were selected through repeated trial-and-error analysis to obtain minimum mean squared error and improved prediction accuracy. The network convergence was monitored based on validation performance, gradient reduction, and stabilization of training error. The Levenberg–Marquardt training algorithm was selected because of its fast convergence capability and effectiveness in nonlinear regression modelling with limited experimental datasets. While RSM effectively establishes statistical relationships and optimization, it is limited in capturing strong nonlinear interactions. ANN overcomes this limitation by learning complex nonlinear mappings, thereby improving prediction accuracy.

## 3. Results and discussion

Results were evaluated between pure diesel (B0) and a dual biodiesel blend of juliflora and kapok (JK B20) with a hydrogen enrichment ratio of 8, 10, and 12 lpm in the CI engine. To improve CI engine characteristics and identify the best emission trade-off, the optimization technique is used to discover the optimal HER.

### 3.1. Brake thermal efficiency

**Fig 3:**
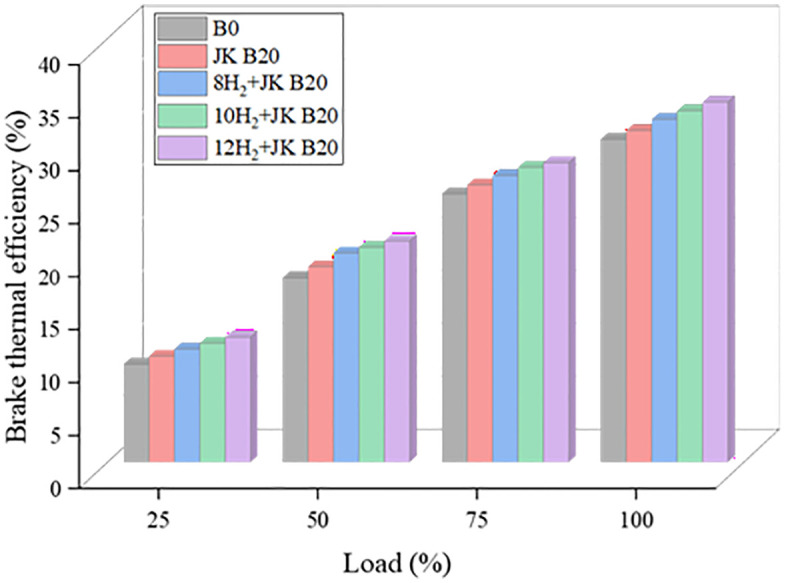
Effect of HER on BTE.

How well heat is transformed into work is determined by BTE. Fig 3 exhibits the cumulative impacts on BTE of the HER and JK dual biodiesel mix B20. Because of the increasing temperatures inside the cylinder, the observations of BTE rise as the load % rises. With a higher oxygen concentration, JK B20 enhances combustion, decreases heat loss, and increases power production, making it more efficient than ordinary diesel [27]. The efficiency of the engine was enhanced by adding H_2_ to the JK B20. In general, the BTE rises in tandem with the HE. When combined with JK B20, a higher HER of 12 lpm can achieve the maximal BTE. Compared to diesel, this method raised the BTE by 11.5%. Thanks to H_2_’s properties including its high CV, rapid burning, and ease of mixing with air BTE has been significantly improved [28]. Furthermore, H_2_ can efficiently infiltrate the entire cylinder due to its short quench distance and fast flame propagation, leading to better combustion and a higher BTE [29]. Although the combined effect of biodiesel oxygen content and hydrogen enrichment improves BTE, their individual contributions were not quantitatively isolated in this study. Biodiesel provides inherent oxygen for improved combustion, while hydrogen enrichment further enhances flame propagation and energy release.

### 3.2. Brake specific fuel consumption

**Fig 4:**
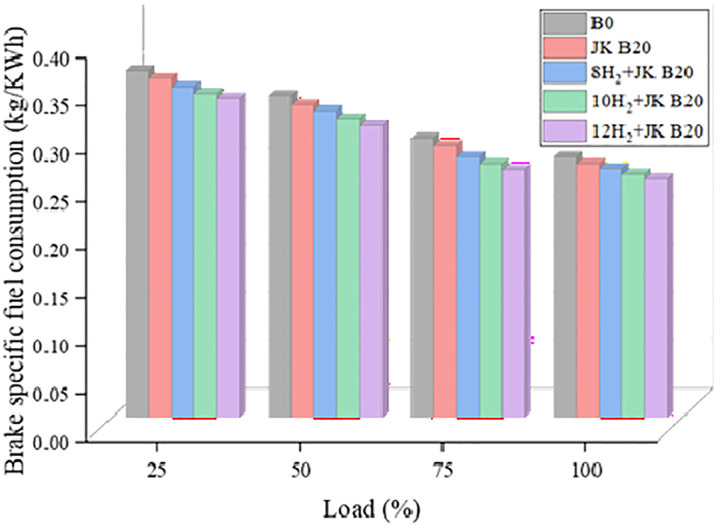
Effect of HER on BSFC.

The mass flow rate to engine power ratio is determined by BSFC. As shown in Fig 4, the BSFC of the CI engine is determined to be 0.272 kg/kWh while operating with diesel at higher loads. The BSFC is found to be lower than B0 while blending JK dual blend (JK B20). One possible explanation is that biodiesel contains a high concentration of oxygen. While the combined effect of H_2_ and dual JK B20 significantly lowered BSFC, blending the two methods had a less impact. At 12 lpm of H_2_, it is possible to see that the BSFC drops as the HER rises with JK B20. When 12H_2_ + JK B20 are used together, they reduce BSFC by as much as 8.5% compared to B0. Despite hydrogen being inducted through the intake manifold, its high diffusivity and flame speed significantly enhance premixed combustion, resulting in more efficient energy conversion and reduced fuel consumption [30]. As a result of H_2_’s greater heating value (HHV), BSFC has decreased. Less fuel is needed to provide the same level of power when using fuel with a reduced igniting delay and a higher HHV [31]. In other words, higher power produced with the aid of H_2_ enrichment for the same amount of fuel could be reason behind this.

### 3.3. Peak cylinder pressure

**Fig 5:**
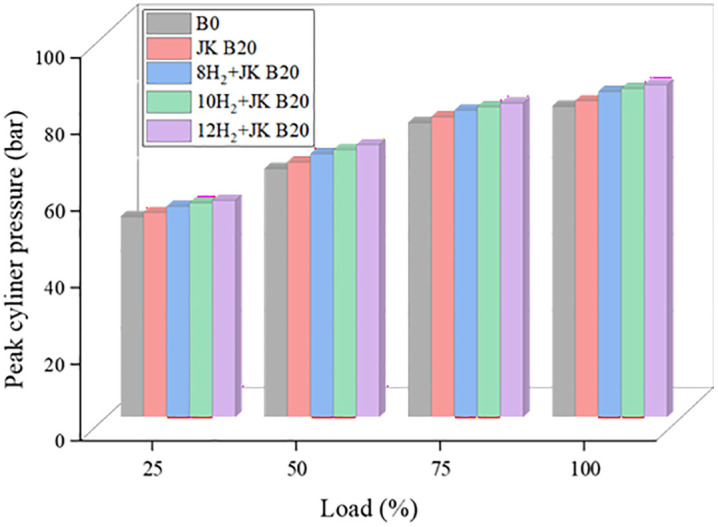
Effect of HER on CP.

The maximum pressure that can be achieved inside the cylinder, or peak CP, is one measure of how well fuel combustion is performing. Fig 5 shows the peak CP plotted against various operating circumstances. At full load, the combustion characteristic results show that the injected fuel amount rose, leading to an increase in CP values. Using HE, JK B20 achieves the highest CP values across the board for engines. The improved physiochemical properties of the blended fuel directly affect the combustion parameters of the CI engine. While H_2_ enrichment boosted CP by up to 6.9% relative to diesel, mixing JK B20 only enhanced it by 2% at full load. Possible explanations for the higher peak CP attainment include H_2_’s early state of charge, stronger diffusivity, and chemical reactivity. Hydrogen enrichment significantly alters the combustion mechanism by increasing the laminar flame speed and reducing ignition delay, which promotes rapid premixed combustion. This results in shorter combustion duration and a higher rate of pressure rise inside the cylinder. The high diffusivity of hydrogen improves mixture homogeneity and accelerates combustion intensity, thereby increasing peak cylinder pressure. This leads to a higher heat release rate during the initial stages of combustion, thereby increasing the rate of pressure rise. Furthermore, the higher adiabatic flame temperature of hydrogen contributes to elevated in-cylinder temperatures, which directly increases peak cylinder pressure. Many investigations have found that HE produces comparable results. The high heat of combustion of H_2_ increases the CP by increasing the internal temperature of the cylinder [32].

### 3.4. Peak heat release rate

**Fig 6:**
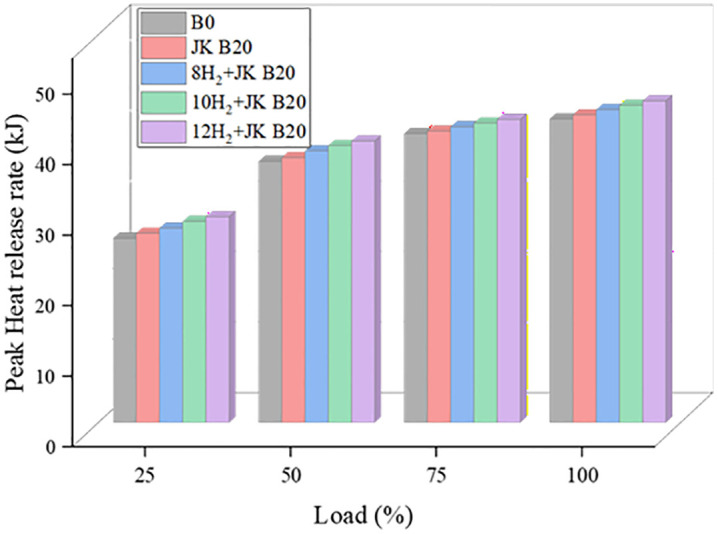
Effect of HER on HRR.

Fig 6 shows that the HRR is significantly affected by the HER and dual JK B20. The HRR foretells the combustion behaviour of the CI engine. As the load grew, the HRR rose as a result of combustion dominance caused by higher in-cylinder pressure and temperature. The JK B20 dual biodiesel blend had a 2% greater HRR than B0 at full load due to its HHV. The HRR was 5.9% higher than B0 and nearly three times higher than JK B20 after H_2_ enrichment. Hydrogen enrichment is also expected to reduce ignition delay due to its low ignition energy and high flame speed. This reduction enhances the premixed combustion phase, resulting in higher peak heat release rates and cylinder pressures. Although ignition delay was not directly measured in the present study, the observed combustion trends and previous literature support this inference [3]. Hydrogen enrichment is also expected to reduce ignition delay due to its low ignition energy and high flame speed. This reduction enhances the premixed combustion phase, resulting in higher peak heat release rates and cylinder pressures.

Consequently, the diffusion combustion phase is shortened due to improved fuel-air mixing and faster combustion kinetics. Consequently, the use of H_2_ is becoming increasingly common, as is further demonstrated by the engine’s higher HRR. It has also been noted that, due to the direct correlation between HER and its HHV, higher HER leads to higher HRR. Control combustion duration and ignition quality were both enhanced by biodiesel’s high cetane number. This increase in HRR is directly associated with the higher peak cylinder pressure observed, as faster energy release during the premixed combustion phase results in a steeper pressure rise. Furthermore, biodiesel’s oxygen concentration causes combustion to be unexpectedly accelerated, leading to a greater maximum CP value for B20 than diesel.

### 3.5. HC emission

**Fig 7:**
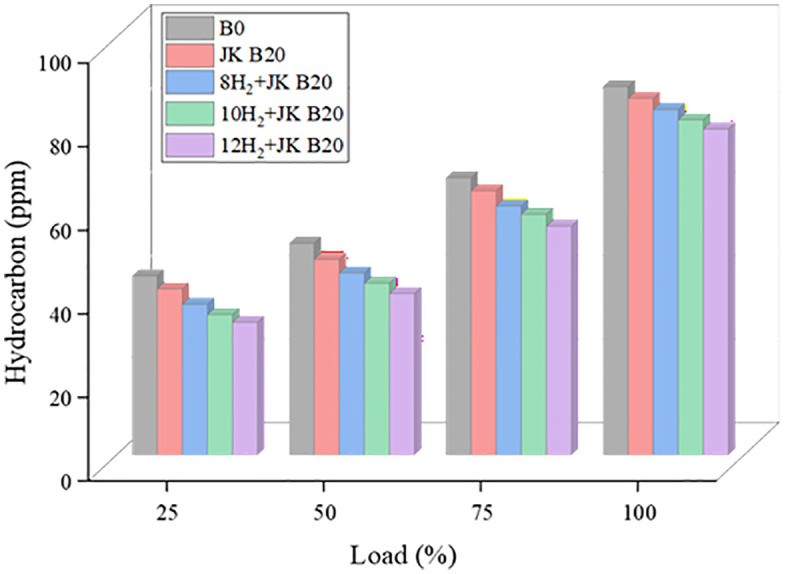
Effect of HER on HC.

Lower cylinder temperatures and incomplete combustion are the main causes of unburned HC production. The increased oxygen content of biodiesel makes it a more efficient fuel to burn. In Fig 7, we can see the HC emissions from the JK B20 dual biodiesel blend and HER. Combining biodiesel with H_2_ to improve the air fuel mixture, increase temperature, and promote post-flame oxidation is a win-win. An 11% drop in HC production relative to B0 is the outcome of increasing the HER, which makes the JK B20 blend burn more efficiently. Because H_2_ improves combustion, HC emissions have been steadily declining. Because of its high flame speed, the fuel-air mixture in the cylinder burns more rapidly and completely, resulting in less fuel that goes to unburned. H_2_ also has lower HC-generating misfire and partial burn rates because of its wider flammability limitations, which can lead to more stable combustion [33].

### 3.6. CO emission

**Fig 8:**
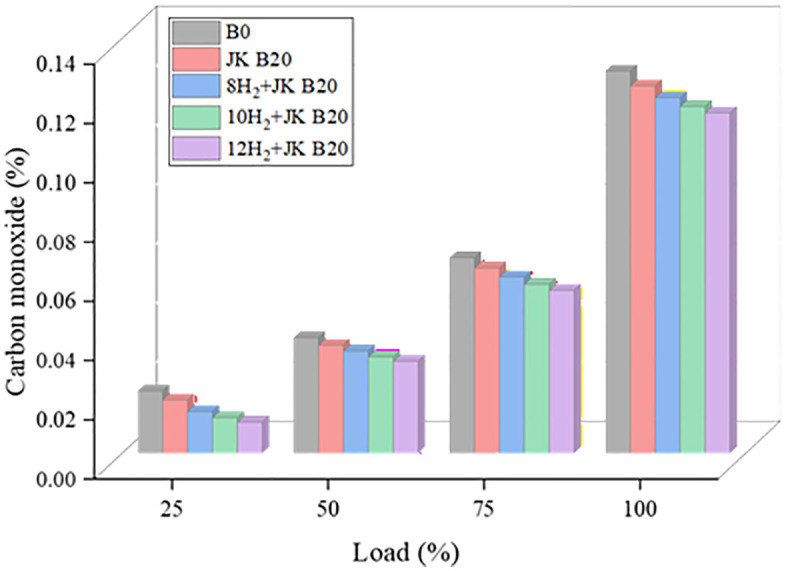
Effect of HER on CO.

Combustion in an oxygen-depleted atmosphere results in the production of carbon monoxide. When fuel combustion does not have enough time at maximum load, the CO emission value suddenly increases. Fig 8 shows how the CO levels of the test mixes changed with load for different HER. HER increased was accompanied by a decline in CO emissions. At 12 lpm of H_2_, the maximum CO emission with JK B20 was 10.9%, compared to regular diesel. The absence of carbon molecules in H_2_ causes a drop in CO, which causes this to happen. The higher oxygen molecular composition of dual biodiesel enhances the availability of oxygen for burning, which in turn reduces CO emissions. The increased C-H ratio and decreased cylinder temperatures commonly found in diesel engines are the main causes of CO emissions from these engines. HER enhances combustion by self-burning, and the lack of carbon in H_2_ could potentially lead to a decrease in CO emissions.

### 3.7. Smoke opacity

**Fig 9:**
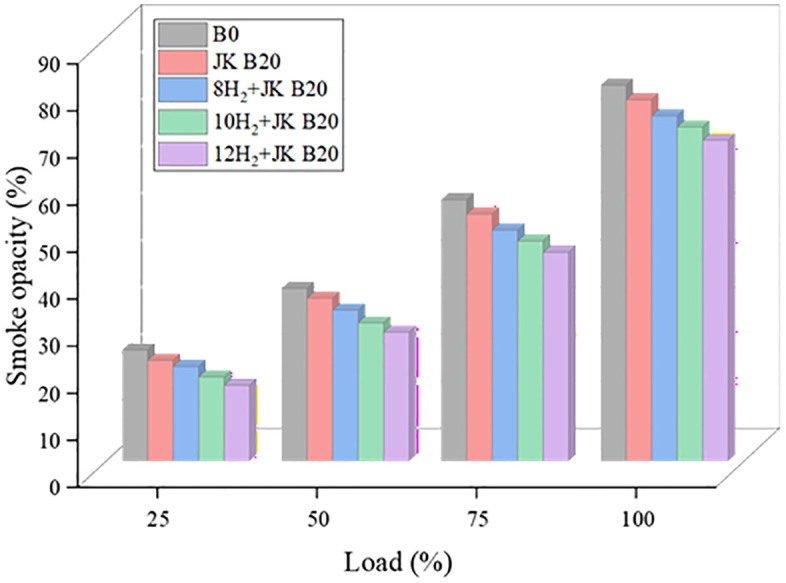
Effect of HER on Smoke.

The by-product of a combustion in a low-oxygen, high-pressure setting is smoke. Fig 9 highlights how HER and JK B20 impact smoke emissions. Smoke emissions development is dependent on biodiesel physiochemistry, air shortage, fuel atomization, and air-fuel mixing. Because of its higher oxygen content, biodiesel burns more efficiently and produces less smoke than diesel. Having said that, the percentage of reduction is lower, the smoke output was drastically cut down as HER approached. At a higher HER of 12 lpm and maximum load, the mixture of JK B20 and diesel reduced smoke by 14.5 percent. Biodiesel blends reduce smoke emissions because of fuel-borne oxygen, which is a property of biodiesel itself. There will be less smoke and particulate matter emitted since the fuel can be burned more completely due to the oxygen concentration [34]. Because H_2_ fuel contains very little sulphur and no aromatic hydrocarbons, its enrichment might significantly reduce smoke emissions. The reduction in CO, HC, and smoke emissions is supported by enhanced oxidation kinetics due to hydrogen addition, which promotes active radical formation (H, O, OH) and faster combustion. Additionally, the inherent oxygen content of biodiesel improves oxidation of carbon species, while the absence of carbon in hydrogen suppresses soot formation.

### 3.8. NOx emission

**Fig 10:**
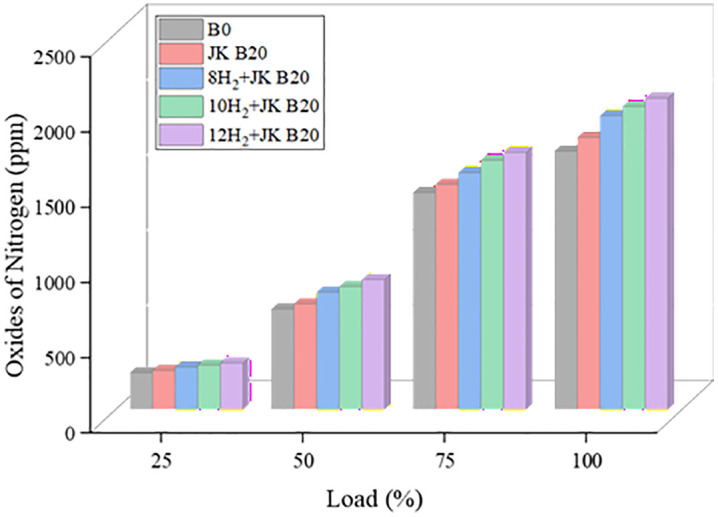
Effect of HER on NOx.

The changes in NOx emission as a function of load and HER are illustrated in Fig 10. As HER rose, the levels of NOx were observed to grow steadily. The oxygen content and the evolved temperature of the combustion chamber are the primary factors that affect the NOx concentrations that are created. There is a direct correlation between the rate of heat release and the rate of H_2_ supply; when the former is increased, NOx emissions are likewise increased. Results showed a 5% increase in NOx with JK B20 and a 20% rise with 12H_2_ + JK B20. Although in-cylinder temperature was not directly measured, the increase in NOx emissions is attributed to higher combustion temperatures inferred from increased heat release rate and peak cylinder pressure. Because of its larger cetane number and elevated oxygen content, JK B20 burns more quickly and reaches higher peak temperatures, which increases NOx production. Consequently, NOx emissions rise [35]. Adding H_2_ to a mixture can make the flames burn faster and more intense, which can raise the peak temperature. Biodiesel encourages full combustion, which could lead to higher combustion temperatures, due to its higher oxygen content compared to normal diesel. Because of this, biodiesel and H_2_ work together to produce less nitrogen oxides (NOx) than either fuel alone. NOx emissions could be further mitigated by implementing control strategies such as exhaust gas recirculation (EGR), injection timing retardation, or water injection, which were not considered in the present study.

### 3.9. RSM Optimization

**Fig 11:**
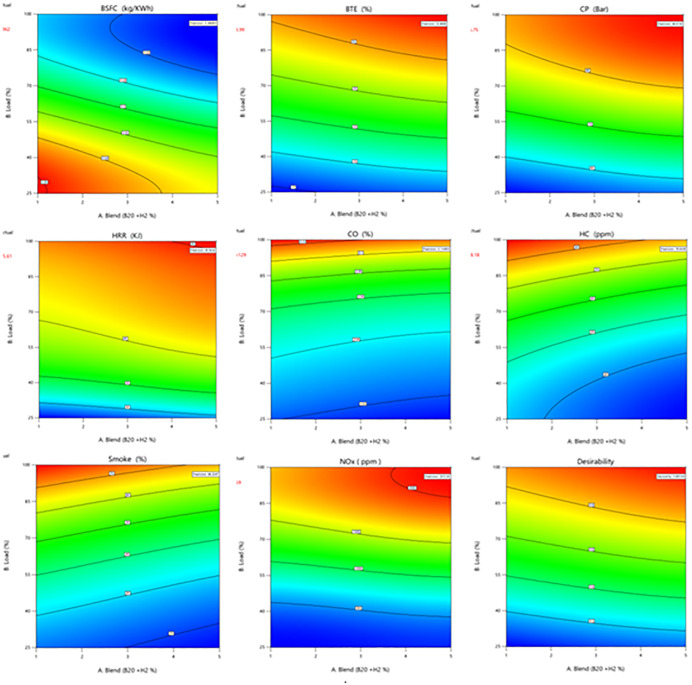
Desirability contour plots.

The RSM model is assessed by comparing expected and actual data. Table 4 shows the data set for numerical analysis. RSM-based regression analysis shows that Hydrogen percentage behaved as expected experimentally. All response variable regression models have assessed and listed as the following. ANOVA was used to assess model performance and critical factors. Table 5 ANOVA data are utilized to create an engine reaction regression model. These factors exerted varying degrees of influence on the response variable as the significant regression models results indicates. An adj. R^2^ value illustrates how H_2_ proportion impacts biodiesel power plant efficiency and confirms correlation coefficients. Cubic model matched to data achieved highest correlation and lowest standard deviation. R^2^ evaluates regression model predictions against data. Prior R^2^ analysis research defined acceptable matches as models with R^2^ values exceeding 0.8. The study found greater R^2^ values, indicating perfect match. Multi-response optimization uses RSM. The optimization criteria reduced BSFC, increased BTE, and enhanced other responses. Desirability results in the Fig 11 demonstrates that the B20 with 12% H_2_ concentration test blend solution 100% load had a high desirability index of 0.995, proving its robustness. Model consistency is shown by the estimated peak CP (86.75 bar) being 0.0016% different from the observed value (86.61 bar). The estimated HRR (45.54 J/°CA) was 0.15% off the measured result (45.61 J/°CA). BTE was 33.87%, close to the experimental number of 33.99%, showing efficient biodiesel with H_2_ mix combustion under ideal conditions [36]. It should be noted that the desirability index is sensitive to the assigned weighting factors and optimization criteria. Variations in parameter importance may lead to different optimal solutions and desirability values. Similar trends occurred in emissions. RSM anticipated both to be close to experimental values. The regression model’s brake specific fuel usage (0.249 kg/kWh) matched experimental results, showing its reliability. Experimental results confirmed RSM predictions, with relative errors < 1% for all responses. The model’s reliability and efficiency and emission optimization of biodiesel–diesel blends are proven.

**Table 4:** Data set of engine outcomes for RSM and ANN Analysis.

std. order	Run order	Load (%)	H_2_ Con. (%)	BSFC (kg/kW.h)	BTE (%)	HC (ppm)	CO (%)	NO_x_ (ppm)	Smoke (%)	CP (bar)	HRR (KJ)
4	1	0	0	0.3617	9.12	52.16	0.0208	248.79	23.46	52.16	26.08
12	2	0	8	0.3371	17.66	65.24	0.0397	670.38	36.91	65.24	37.18
19	3	0	10	0.2908	25.13	76.49	0.0657	1425.78	55.36	76.49	40.77
15	4	0	12	0.2724	30.47	81.2	0.1289	1721.17	79.99	81.2	43.14
17	5	25	0	0.3532	9.96	53.5	0.0176	251.21	21.72	53.5	26.77
2	6	25	8	0.3278	18.59	66.88	0.0367	712.19	34.45	66.88	37.73
6	7	25	10	0.282	26.1	78.27	0.0623	1500.58	52.37	78.27	41.26
1	8	25	12	0.2658	31.42	82.98	0.1243	1822.56	76.69	82.98	43.65
10	9	50	0	0.3453	10.73	54.87	0.0147	275.14	19.83	54.87	27.63
7	10	50	8	0.3189	19.48	68.5	0.0341	769.63	31.95	68.5	38.45
3	11	50	10	0.2736	27.04	79.99	0.0593	1585.11	49.46	79.99	41.92
14	12	50	12	0.2594	32.36	84.63	0.1202	1927.8	73.57	84.63	44.32
5	13	75	0	0.3385	11.36	56.02	0.0122	301.33	17.93	56.02	28.51
13	14	75	8	0.3109	20.23	69.85	0.0321	823.43	29.54	69.85	39.18
18	15	75	10	0.266	27.86	81.39	0.0571	1660.14	46.74	81.39	42.59
9	16	75	12	0.2536	33.2	85.93	0.1169	2017.64	70.74	85.93	45
20	17	100	0	0.3332	11.74	56.7	0.0104	310.53	16.13	56.7	29.25
8	18	100	8	0.3043	20.76	70.69	0.0309	854.36	27.33	70.69	39.77
16	19	100	10	0.2596	28.49	82.23	0.0556	1706.4	44.33	82.23	43.11
11	20	100	12	0.2489	33.87	86.62	0.1146	2072.83	68.33	86.62	45.54

**Table 5:** ANNOVA.

Model	NO_x_	HC	CO	Smoke	HRR	CP	BTE	BSFC
Std. Dev.	13.34	0.2435	0.0005	0.2499	0.1390	0.3554	0.2174	0.0014
Mean value	1132.85	56.04	0.0577	43.84	38.09	71.71	22.28	0.3001
C.V. %	1.18	0.4345	0.8004	0.5699	0.3650	0.4956	0.9759	0.4621
R²	0.9998	0.9999	0.9999	0.9999	0.9998	0.9995	0.9995	0.9993
Predicted R²	0.9996	0.9998	0.9999	0.9999	0.9996	0.9991	0.9993	0.9986
Adjusted R²	0.9992	0.9996	0.9996	0.9997	0.9987	0.9978	0.9990	0.9976
F-value	5314.4	12311.8	16708.6	15150.8	4788.22	2371.7	5748.8	1511.8
p-value	<0.0001	<0.0001	<0.0001	<0.0001	<0.0001	<0.0001	<0.0001	<0.0001

Regression equations


$$\begin{array}{cc} & 𝐍𝐎𝐱\;=\text{1186}.\text{86}+\text{143}.\text{42A}+\text{1272}.\text{84 B}+\text{72}.\text{48 AB}-\text{13}.\text{14 }{\text{A}}^{\text{2}}-\text{85}.\text{39 }{\text{B}}^{\text{2}}-\text{17}.\text{66 }{\text{A}}^{\text{2}}\text{B}-\text{14}.\text{40 }\\ & {\text{AB}}^{\text{2}}-\text{25}.\text{67 }{\text{A}}^{\text{3}}-\text{446}.\text{51 }{\text{B}}^{\text{3}} \end{array}$$

$$\begin{array}{cc} & 𝐒𝐦𝐨𝐤𝐞=\text{ 39}.\text{95}-\text{5}.\text{36 A}+\text{26}.\text{19 B }-\text{1}.\text{08 AB}+\text{0}.\text{2771 }{\text{A}}^{\text{2}}+\text{6}.\text{75 }{\text{B}}^{\text{2}}+\text{0}.\text{3146 }{\text{A}}^{\text{2}}\text{B}+\text{0}.\text{4568 }{\text{AB}}^{\text{2}}\\ & \;\;+\text{0}.\text{1600 }{\text{A}}^{\text{3}}+\text{0}.\text{6773 }{\text{B}}^{\text{3}} \end{array}$$

$$\begin{array}{cc} & 𝐇𝐂\;=\;+\text{50}.\text{88}-\text{5}.\text{98 A}+\text{24}.\text{54 B}+\text{0}.\text{3072 AB}+\text{0}.\text{7914 }{\text{A}}^{\text{2}}+\text{8}.\text{58 }{\text{B}}^{\text{2}}-\text{0}.\text{3497 }{\text{A}}^{\text{2}}\text{B}+\text{0}.\text{5288 }\\ & {\text{AB}}^{\text{2}}+\text{0}.\text{0717 }{\text{A}}^{\text{3}}-\text{1}.\text{33 }{\text{B}}^{\text{3}} \end{array}$$

$$\begin{array}{cc} & 𝐂𝐎\;=\;+\text{0}.\text{0442}-\text{0}.\text{0048 A}+\text{0}.\text{0359 B}-\text{0}.\text{0010 AB}+\text{0}.\text{0012 }{\text{A}}^{\text{2}}+\text{0}.\text{0233 }{\text{B}}^{\text{2}}+\text{0}.\text{0003 }{\text{A}}^{\text{2}}\text{B}-\text{0}.\text{0017 }\\ & {\text{AB}}^{\text{2}}+\text{0}.\text{0002 }{\text{A}}^{\text{3}}+\text{0}.\text{0169} \end{array}$$

$$\begin{array}{cc} & 𝐇𝐑𝐑=\;+\text{40}.\text{71}+\text{1}.\text{42 A}+\text{4}.\text{82 B}-\text{0}.\text{1923 AB}+\text{0}.\text{0264 }{\text{A}}^{\text{2}}-\text{4}.\text{73 }{\text{B}}^{\text{2}}-\text{0}.\text{0056 }{\text{A}}^{\text{2}}\text{B}+\text{0}.\text{1789 }\\ & {\text{AB}}^{\text{2}}-\text{0}.\text{2050 }{\text{A}}^{\text{3}}+\text{3}.\text{53 }{\text{B}}^{\text{3}} \end{array}$$

$$\begin{array}{cc} & 𝐂𝐏\;=\text{ 74}.\text{80}+\text{3}.\text{16 A}+\text{17}.\text{53 B}+\text{0}.\text{2181 AB}-\text{0}.\text{5807 }{\text{A}}^{\text{2}}-\text{5}.\text{05 }{\text{B}}^{\text{2}}-\text{0}.\text{1427 }{\text{A}}^{\text{2}}\text{B}-\text{0}.\text{3499 }\\ & {\text{AB}}^{\text{2}}-\text{0}.\text{3233 }{\text{A}}^{\text{3}}-\text{2}.\text{65 }{\text{B}}^{\text{3}} \end{array}$$

$$\begin{array}{cc} & 𝐁𝐓𝐄\;=\;+\text{23}.\text{47}+\text{1}.\text{75 A}+\text{11}.\text{40 B}+\text{0}.\text{1946 AB}-\text{0}.\text{2475 }{\text{A}}^{\text{2}}-\text{1}.\text{93 }{\text{B}}^{\text{2}}+\text{0}.\text{0598 }{\text{A}}^{\text{2}}\text{B}-\text{0}.\text{1277 }\\ & {\text{AB}}^{\text{2}}-\text{0}.\text{1150 }{\text{A}}^{\text{3}}-\text{0}.\text{5901 }{\text{B}}^{\text{3}} \end{array}$$

$$\begin{array}{cc} & 𝐁𝐒𝐅𝐂\;=\;\text{0}.\text{2955}-\text{0}.\text{0170 A}-\text{0}.\text{0710 B}+\text{0}.\text{0013 AB}+\text{0}.\text{0017 }{\text{A}}^{\text{2}}+\text{0}.\text{0069 }{\text{B}}^{\text{2}}-\text{0}.\text{0004 }{\text{A}}^{\text{2}}\text{B}+\text{0}.\text{0034 }\\ & {\text{AB}}^{\text{2}}+\text{0}.\text{0007 }{\text{A}}^{\text{3}}+\text{0}.\text{0280 }{\text{B}}^{\text{3}} \end{array}$$

### 3.10. ANN modelling

**Fig 12:**
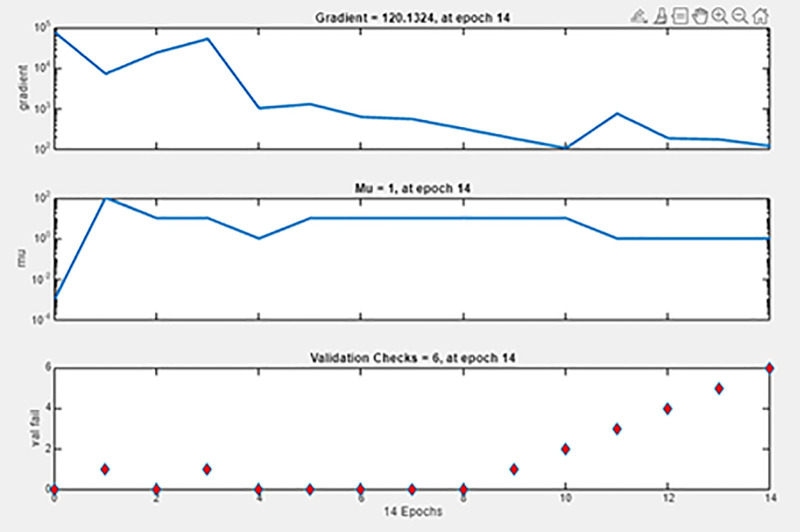
Training state plot.

**Fig 13:**
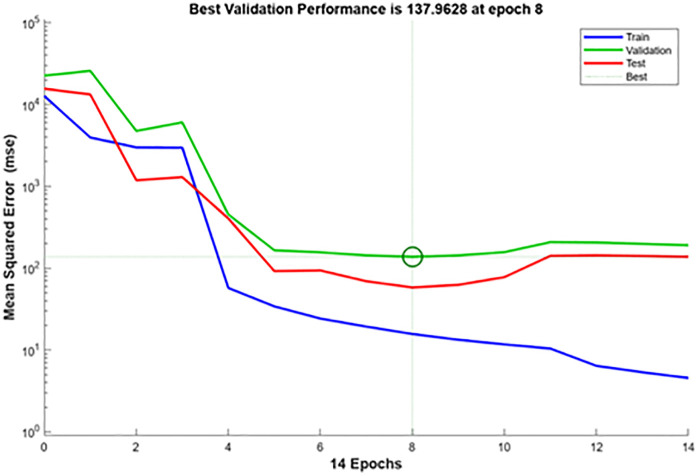
Training performance curve.

**Fig 14:**
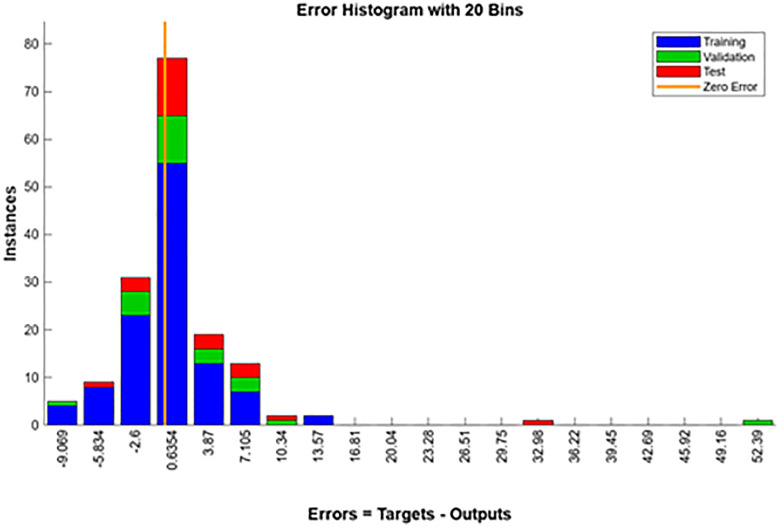
Error histogram.

The ANN model, created using MATLAB’s Neural Network Fitting Tool, predicted CI engine performance under different fuel blends and loads. A five-layer design with an input, three hidden, and output layer was used for model creation and validation. The dataset was split 70% training, 15% validation, and 15% testing for equitable learning and evaluation. Randomly divided data included 14 training, 3 validations, and 3 testing samples. The model demonstrated good predictive capabilities and outstanding pattern learning with high correlation values (R = 0.9999) across all datasets. Due to small dataset size, validation MSE is greater than training Mean squared error (MSE), which is 15.73, suggesting minor overfitting. Overall, the ANN performs well for small-sample nonlinear regression. Nonlinear relationships between engine load, biodiesel mix ratio, and performance parameters were recorded using the network design. Fig 12 illustrates sustained gradient reduction and consistent learning in the training state plot. The Fig 13 training performance curve indicates good convergence and validation with 137.96 MSE accuracy across 8 epochs. Small differences between training, validation, and testing curves indicate model resilience. Fig 14’s error histogram shows little variance and strong fitting accuracy with most errors around zero. The ANN model was validated using statistical indicators such as coefficient of determination (R), mean squared error (MSE), and average relative error (ARE), confirming high prediction accuracy and reliability [37].

**Fig 15:**
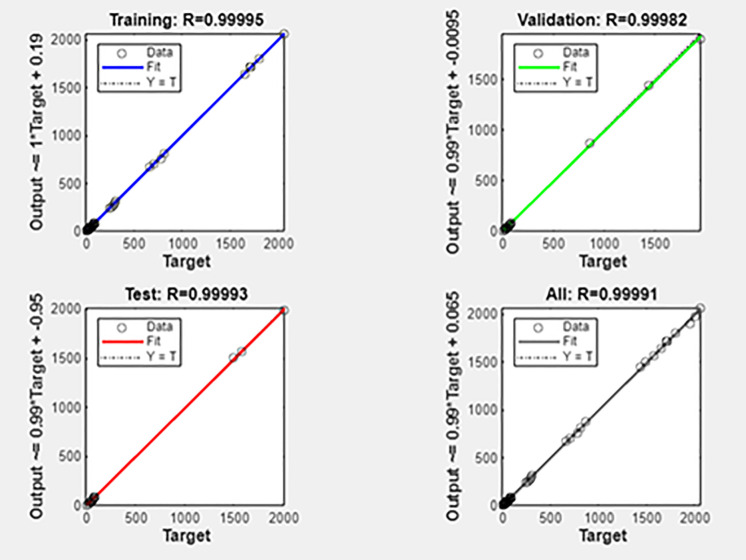
Regression plot.

The regression curve between experimental target values and ANN-predicted outputs for training, validation, and testing datasets is shown in Fig 15. Data points close to the regression line show a strong linear relationship and minimum variance between observed and projected engine performance characteristics. An ANN model with a correlation coefficient (R) around unity (0.99–1.00) predicts good accuracy and reliability. The network’s near-perfect alignment across all datasets captures the nonlinear mapping between input parameters and output responses, proving the model’s resilience and applicability. The strong agreement between predicted and experimental results with R values close to unity indicates excellent generalization and minimal overfitting. All results confirm and match the ANN model with experimental data, making it a reliable and efficient prediction framework for CI engine characteristics optimization.

### 3.11. Comparison of ANN and RSM

Mean Squared Error (MSE), Absolute Relative Error (ARE) in percentage, and R-squared values were evaluated and connected to better understand the ANN and RSM model’s predictions. RSM model has lower error rates than ANN model, indicating improved data fitting and evaluation precision. Due to its ability to measure engine performance and emissions, the RSM model forecasts outcomes more accurately. In contrast, ANN predicts higher engine response R^2^, MSE, and ARE. Consequently, it can the RSM model accurately predicted engine reactions across a wide range of factors, including engine load and H_2_ concentration, both inside and outside the studied parameters.

## 4. Conclusion

The characteristics of CI engine fuelled with dual JK biodiesel blend of B20 along with the approach of hydrogen enrichment has been studied. ANN complements RSM by addressing its limitation in modeling nonlinear behaviour, providing improved predictive performance for complex engine response characteristics. The following results were obtained.

- The combined effect of 12H_2_ + JK B20 resulted in higher BTE, CP and HRR by 11.5%, 6.9% and 5.9% as compared to diesel. Also, there was a 8.5%, 10.9%, 11.5% and 14.6% reduction in BSFC, CO, HC and smoke emission were achieved as the effect of better combustion with H_2_ and JK B20. As a trade-off higher NOx emission has been noticed.
- The RSM results shows that the test blend B20 with 12% H_2_ at 100% load had a high desirability index of 0.995. ANN model was validated and fitted with experimental data, making it a reliable and efficient prediction framework for CI engine characteristics optimization.
- RSM model shows reduced rates of error than ANN model, thereby signifying enhanced precision regarding both data fitting and evaluation capabilities. ANN predicts engine reactions in real-time similarly to RSM.
- The scalability of hydrogen-enriched dual biodiesel systems depends on advancements in hydrogen production, storage, and infrastructure. While biodiesel is readily deployable, hydrogen usage currently faces logistical challenges, which are expected to be improved with the development of green hydrogen technologies.

**Fuel blends**

B0: Pure diesel (100%)
JK B20: Juliflora kapok biodiesel (20%) + diesel (80%)
JK B100: Juliflora biodiesel (50%) + kapok biodiesel (50%)
8H_2_+JK B20: Hydrogen (08 lpm) + JK B20 (20%) + diesel (80%)
10H_2_+JK B20: Hydrogen (10 lpm) + JK B20 (20%) + diesel (80%)
12H_2_+JK B20: Hydrogen (12 lpm) + JK B20 (20%) + diesel (80%)

## Abbreviations

ANN: Artificial neural network
BTE: Brake thermal efficiency
BSFC: Brake specific fuel consumption
CI: Compression ignition
CO: Carbon monoxide
CP: Cylinder pressure
HC: Hydrocarbon
HE: Hydrogen enrichment
HER: Hydrogen enrichment ratio
HRR: Heat release rate
NO_x_: Nitrogen oxides
RSM: Response surface methodology
